# Supplementary material for: Development of a computer-aided design software for the quantitative evaluation of aesthetic damage
Source: PLoS One. 2019 Dec 18;14(12):e0226322. doi: 10.1371/journal.pone.0226322 (PMC6919621; doi:10.1371/journal.pone.0226322)
Supplement: S1 Code site — 1. (ZIP) [file pone.0226322.s001.zip › [EESC_jr][Projeto]Relatorio_07_21/reports/templates_zip/gentelella-master/vendors/morris.js/examples/non-continuous.html]

# Non-continuous data

Null series values will break the line when rendering, missing values will be skipped

```
/* data stolen from http://howmanyleft.co.uk/vehicle/jaguar_'e'_type */
var day_data = [
  {"period": "2012-10-01", "licensed": 3407},
  {"period": "2012-09-30", "sorned": 0},
  {"period": "2012-09-29", "sorned": 618},
  {"period": "2012-09-20", "licensed": 3246, "sorned": 661},
  {"period": "2012-09-19", "licensed": 3257, "sorned": null},
  {"period": "2012-09-18", "licensed": 3248, "other": 1000},
  {"period": "2012-09-17", "sorned": 0},
  {"period": "2012-09-16", "sorned": 0},
  {"period": "2012-09-15", "licensed": 3201, "sorned": 656},
  {"period": "2012-09-10", "licensed": 3215}
];
Morris.Line({
  element: 'graph',
  data: day_data,
  xkey: 'period',
  ykeys: ['licensed', 'sorned', 'other'],
  labels: ['Licensed', 'SORN', 'Other'],
  /* custom label formatting with `xLabelFormat` */
  xLabelFormat: function(d) { return (d.getMonth()+1)+'/'+d.getDate()+'/'+d.getFullYear(); },
  /* setting `xLabels` is recommended when using xLabelFormat */
  xLabels: 'day'
});
```
